# Design, Synthesis, and Biological Evaluation of a New Series of Biphenyl/Bibenzyl Derivatives Functioning as Dual Inhibitors of Acetylcholinesterase and Butyrylcholinesterase

**DOI:** 10.3390/molecules22010172

**Published:** 2017-01-20

**Authors:** Dong-mei Wang, Bo Feng, Hui Fu, Ai-lin Liu, Lin Wang, Guan-hua Du, Song Wu

**Affiliations:** 1 State Key Laboratory of Bioactive Substances and Functions of Natural Medicines & Ministry of Health Key Laboratory of Biosynthesis of Natural Products, Institute of Materia Medica, Chinese Academy of Medical Sciences & Peking Union Medical College, Beijing 100050, China; wangdmchina@imm.ac.cn (D.-m.W.); fengbo@imm.ac.cn (B.F.); liuailin@imm.ac.cn (A.-l.L.); wlin@imm.ac.cn (L.W.); dugh@imm.ac.cn (G.-h.D.); 2 Beijing Institutes For Drug Control, Beijing 100050, China; Fuhuifree@sina.com

**Keywords:** Alzheimer’s disease, biphenyl/bibenzyl derivatives, acetylcholinesterase inhibitors, butyrylcholinesterase inhibitors, molecular docking

## Abstract

Alzheimer’s disease (AD), the most common form of dementia in adults, is a progressive neurodegenerative disorder of the brain characterized by loss of memory and steady deterioration of cognition. Here, a series of symmetrical molecules containing biphenyl/bibenzyl scaffolds (**12**–**36**) were designed, synthesized, and evaluated for their ability to inhibit both acetylcholinesterase (AChE) and butyrylcholinesterase (BuChE). A biological evaluation showed that most of these biphenyl derivatives were potent AChE and BuChE inhibitors. Among them, compound **15** displayed the greatest ability to inhibit BuChE (IC_50_ = 0.74 µM) and was also a good AChE inhibitor (IC_50_ = 1.18 µM). Compound **19** was not only a potent AChE inhibitor (IC_50_ = 0.096 µM), but also a mild BuChE inhibitor (IC_50_ =1.25 µM). Overall, these results suggested that compound **19** may be a promising agent in the treatment of AD.

## 1. Introduction

Alzheimer’s disease (AD), the most common form of dementia in adults, is a progressive neurodegenerative disorder of the brain characterized by loss of memory and steady deterioration of cognition. This disease currently affects more than 30 million people worldwide [1]. However, the exact pathophysiology of AD remains unclear. Several findings indicate that amyloid-β plaques [2], tau protein aggregation [3], oxidative stress [4,5], and a low level of acetylcholine in the brain play important roles in the pathophysiology of the disease [6]. At present, no drug is available to decrease, reverse, or stop the pathological process of AD. Although several research strategies have been envisaged recently, the current therapeutic option is limited to only four AChE inhibitors [7,8], namely, donepezil, rivastigmine, galantamine, and tacrine (now discontinued), and one *N*-methyl-d-aspartate receptor antagonist, memantine [9].

However, two types of cholinesterase (ChE) enzymes have been found in the central nervous system, AChE and BuChE. Studies have indicated that BuChE catalyzes the hydrolysis of acetylcholine in the brains of normal people, as well as patients with Alzheimer’s disease (AD) [10]. While ACh activity decreases to 10%–15% of normal values in certain brain regions in patients with mild to severe AD, BuChE levels remain unchanged, or even increase, with disease progression [11]. Therefore, mixed AChE/BuChE inhibitors may result in higher efficacy for treatment of AD. Rivastigmine, used in AD treatment, is a potent inhibitor of both enzymes [12]. Selective BuChE inhibitors cause an elevation of acetylcholine, augment long-term potentiation, and enhance learning. They have also been shown to decrease amyloid-β in a rodent model of AD [13]. Therefore, dual AChE/BuChE inhibitors may have better clinical efficacy without remarkable adverse effects.

**Figure 1:**
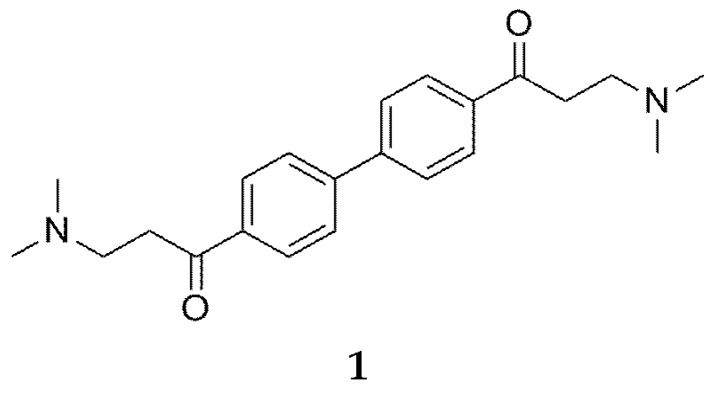
Chemical Structure of **1**.

To find new ChE inhibitors, structure-based virtual screening and high throughput screening were used in the present study to screen the Institute of Meteria Medica’s library of compounds, which contains more than 10,000 molecules. A small symmetrical molecule, 4,4′-bi-(β-dimethylamino-propinoyl)-biphenyl (**1**, Figure 1), was discovered to be a novel dual inhibitor of AChE and BuChE, with a concentration required for 50% inhibition of the maximal response in vitro (its IC_50_ value) of 0.75 µM for AChE and 0.19 µM for BuChE.

The biphenyl scaffold is a privileged structure [14] and it exists in 4.3% of the molecules in commercially available drugs [15]. However, only limited studies have examined these types of compounds for their anti-AChE and anti-BuChE activities. Thus, to find potential anti-AD agents, we report here on the synthesis of a series of derivatives and analogues of compound **1**. The chemical modifications included the following: (i) replacing the dimethylamino group with appropriate secondary amines and heterocyclic amines; (ii) changing the length of the linkers that connect the biphenyl carbonyl core with an amine moiety; (iii) alternating the rigid biphenyl core with a flexible bibenzyl; and (iv) reducing the carbonyl group to a hydroxyl group.

## 2. Results and Discussion

### 2.1. Chemistry

**Scheme 1:**
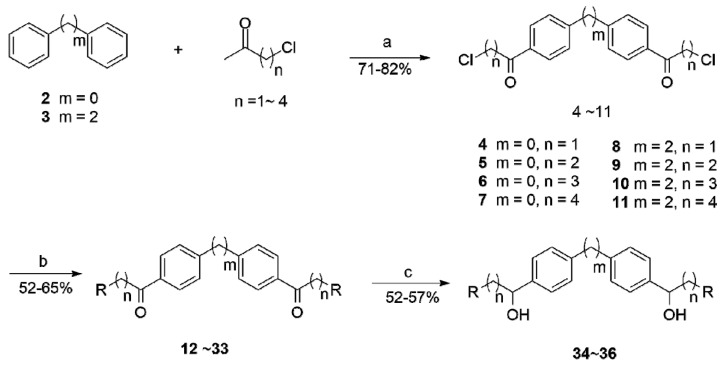
Synthetic route for target compounds **12**–**36**. Reagents, conditions, and yields: (a) Cl(CH_2_)_n_COCl, CS_2_; 40–50 °C, 4–8 h; 71%−82%; (b) secondary amines or heterocyclicamines, CH_3_CN, K_2_CO_3_, KI; 50 °C, 20 min; 52%−65%; (c) NaBH_4_, THF; 50 °C, 6 h; 52%–57%.

The synthetic route for these target compounds (**12**–**36**) is outlined in Scheme 1. Commercially available biphenyl **2** or bibenzyl **3** reacted with Cl(CH_2_)_n_COCl (*n* = 1–4) in the presence of AlCl_3_ in dry CS_2_ at 40–50 °C to generate intermediates (**4**–**11**) with yields of 71%–82%. The target compounds (**12**–**33**) were synthesized by substitution of chlorine in the intermediates (**4**–**11**) with appropriate secondary alkyl amines or heterocyclic amines. The reaction was carried out in CH_3_CN with K_2_CO_3_ and KI. The yields were 52%–65%. Reduction of compounds **19**, **20**, and **30** with sodium borohydride in THF gave the desired alcohols (**34**–**36**) with yields of 52%–57%. Diketones (**19**, **20**, and **30**) are symmetrical compounds, but two asymmetrical carbon centers can be produced during the reduction process to generate compounds **34**–**36**. Thus, the final products may have existed as three isomers, namely, distereomeric mixtures of a meso form (R,S/S,R), and a racemic mixture of two chiral compounds (R,R) and (S,S). These isomers can be separated by chiral column chromatography. However, as our aim is only to investigate whether the carbonyl groups in the molecules were crucial for biological activities, the separation was not conducted. Twenty-one compounds were novel, and the other four compounds have been previously reported [16], although neither the synthetic route nor the structure–activity relationships were provided. The crude products were purified in the present study by column chromatography on silica gel eluted with CH_2_Cl_2_ and MeOH. The purities of all compounds were higher than 99%. The structures of the target compounds are shown in Table 1.

### 2.2. Biological Evaluation and SAR Analysis

The abilities of the target compounds (**12**–**36**) to inhibit AChE (from rat brain) and BuChE (from human serum) activities were evaluated in vitro using the spectrophotometric method of Ellman [16]. Donepezil and tetraisopropyl-pyrophosph-oramide (iso-OMPA) were used as reference compounds, and each test was conducted in triplicate. The IC_50_ values are summarized in Table 1. We found that most of the compounds showed good inhibition against both AChE and BuChE activities, with IC_50_ values ranging from micromolar to sub-micromolar levels. Among these compounds (**12**–**36**), compound **19** showed the strongest inhibition against AChE, with an IC_50_ value of 0.096 µM, approximately half the potency of donepezil (IC_50_ = 0.044 µM). Compound **19** also exhibited potent inhibition of BuChE (IC_50_ = 1.25 µM) which was higher than donepezil. These results indicated that compound **19** was a potent dual inhibitor against AChE and BuChE. We also found that compound **15** exhibited the strongest inhibition against BuChE, with IC_50_ values of 0.74 µM, which was as potent as the reference compound iso-OPMA (IC_50_ = 0.72 µM); additionally, compound **15** was a potent AChE inhibitor (IC_50_ = 1.18 µM).

For all compounds, the length of linker between biphenylcarbonyl or bibenzylcarbonyl and the tertiary amine moiety played a significant role in their inhibitory activities against both AChE and BuChE. The results indicated that a linker length of one carbon atom was not preferred. Compounds with such linker length (**12**–**14**, **25**–**28**) showed no activity, except for compounds **14**, **25**, and **28**. These three compounds displayed low potency for BuChE, with IC_50_ values of 1.44 µM, 9.11 µM, and 6.30 µM, respectively. For the biphenyl derivatives, the biological assay showed that their AChE and BuChE inhibitory activities decreased as the length of linker increased from one carbon atom to three or four carbon atoms, although compound **24**, containing a four-carbon linker, was only slightly less potent (IC_50_ = 0.82 µM for AChE) than compound **19**, with a two-carbon linker (IC_50_ = 0.096 µM for AChE). These results suggested that a linker length of two carbon atoms was optimal for improving AChE inhibitory activities in compounds with a biphenyl moiety.

By contrast, the linker length for the bibenzyl derivatives made little difference with respect to their ability to inhibit AChE activity. For example, bibenzyl derivative **33** (IC_50_ = 0.19 µM for AChE), with a four-carbon linker, displayed similar inhibitory activity to that for compound **19** (IC_50_ = 0.096 µM for AChE), having a two-carbon linker. Furthermore, replacing the dimethylamine moiety in compound **1** with a heterocycloamine, such as piperidine, pyrrolidine, or azetidine, enhanced the inhibition, especially for compound **19**, which was eight-fold more potent than the lead compound (**1**) for inhibiting AChE, while its ability to inhibit BuChE activity was only mildly decreased. Compared with biphenyl derivatives, the corresponding bibenzyl derivatives generally displayed less potent inhibition against both AChE and BuChE. Finally, it appeared that the carbonyl groups in the molecules were indispensable. Reducing compounds **19**, **20**, and **30** to the corresponding alcohols (**34**–**36**) caused them all to lose their ability to inhibit both AChE and BuChE.

### 2.3. Molecular Docking

**Figure 2:**
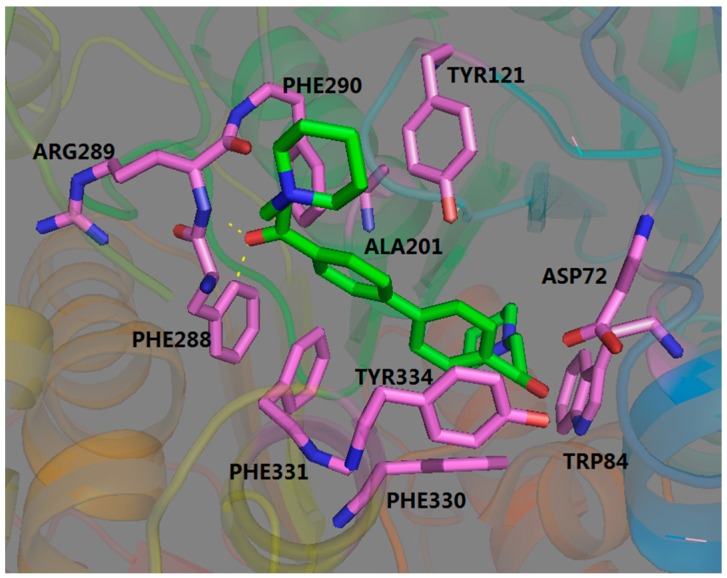
Three-dimensional docking model of compound **19** with AChE.

**Figure 3:**
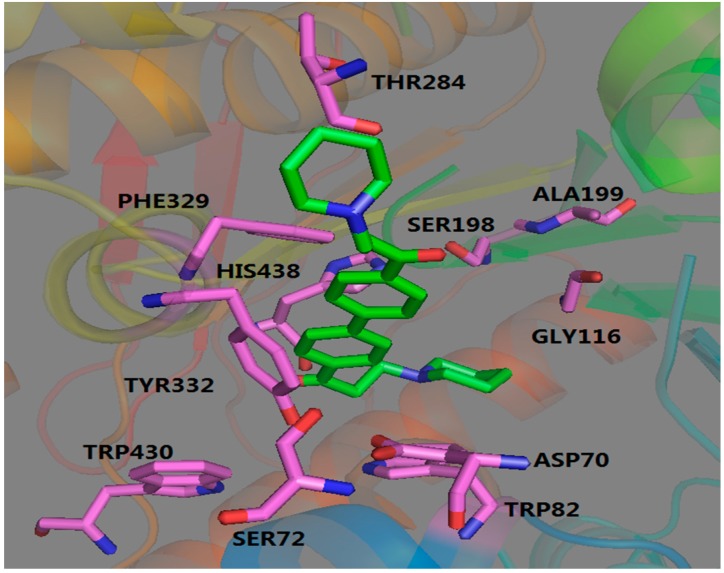
Three-dimensional docking model of compound **19** with BuChE.

To further investigate **19**, the most potent compound against AChE and BuChE, a molecular docking study was performed, and the different types of interactions between **19** and AChE and BuChE are shown in Figure 2 and Figure 3. As shown in Figure 2, compound **19** was located on the long tunnel of the active site interacting with some residues. To be more specific, the aromatic structure of compound **19** adopted an appropriate orientation for its binding to the phenyl ring of TYR334 and PHE330 via π-π stacking interaction. In addition to the hydrophobic interaction, we can also see two key amino residues (PHE288 and ARG289) forming hydrogen bonds with the carbonyl group. All of these results indicated that compound **19** could bind to AChE.

Compare to AChE, BuChE displayed a strong hydrophobic interaction with compound **19**. As shown in Figure 3, the symmetric piperidine ring could insert into the hydrophobic cavities as well. One interaction consisted of HIS438 and TRP82, and the other was formed by THR284, PHE329. Furthermore, the aromatic structure of compound **19** adopted an appropriate orientation for its binding to the phenyl ring of TYR332 via a π-π stacking interaction as well. These results illustrated that compound **19** also can bind to BuChE.

## 3. Materials and Methods

### 3.1. Chemistry

Melting points were measured on a YRT-3 apparatus (Tianjin precision apparatus factory, Tian Jin, China), uncorrected. ^1^H-NMR spectra were recorded on a Varain Mercury-300 & 400 instrument (Varian, Salt Lake City, UT, USA) using tetramethylsilane as an internal standard, DMSO-*d*_6_ and CDCl_3_ as solvents, chemical shifts (δ) in ppm, coupling constants (*J*) in Hz. High-resolution mass spectra were determined with Thermo Scientific Exactive Plus mass spectrometry (Thermo Scientific, Waltham, MA, USA) with the ESI method. Column chromatography was carried out using 200–300 mesh silica gel (Qingdao Marine Chemical Inc., Qingdao, China,). All chemicals and solvents were analytical reagent grade and were used without further purification. All tested compounds were purified until the purity ≥99% detected by HPLC (Shimadzu Co., Kyoto, Japan) under a wavelength of 254 nm, NMR.

#### 3.1.1. General Method for Synthesis of Friedel-Crafts Acylation Intermediates (**4**–**11**)

A mixture of commercial available biphenyl (**2**) or bibenzyl (**3**) (1.0 mmol) and AlCl_3_ (3.0 mmol) was stirred in dry CS_2_ (60 mL), and followed by Cl(CH_2_)_n_COCl (*n* = 1–4) (3.0 mmol) was added drop-wise. The reaction mixture was stirred at 50–80 °C for 4–8 h. Then the reaction mixture was poured into 200 mL ice water, and the solid compound was precipitated. After washing with cold ethanol, the solid was dried to afford compounds (**4**–**11**) with the yield of 71%–82%.

#### 3.1.2. General Method for Synthesis of the Target Compounds (**12**–**33**)

A mixture of secondary alkyl amines or heterocyclic amines (3.0 mmol), anhydrous K_2_CO_3_ (4.0 mmol) and KI (1.0 mmol) was stirred in acetonitrile (40 mL) at 50 °C for 30 min. Then the Friedel-Crafts acylation intermediates (**4**–**11**) was added and stirred for 20 min. The reaction mixture was filtered and concentrated. After washing with water, the mixture was filtered and subjected to silica gel column chromatography with CH_2_Cl_2_:MeOH = 100:1 as an eluent to afford the target compounds (**12**–**33**) with the yield of 52%–65%.

*1,1′-([1,1′-Biphenyl]-4,4′-diyl)bis(2-(dimethylamino)ethanone)* (**12**). Yield, 52%; m.p. 113.7–114.6 °C; ^1^H-NMR (300 MHz, CDCl_3_) δ 8.10 (d, *J* = 8.1, 4H), 7.70 (d, *J* = 8.1, 4H), 3.79 (s, 4H), 2.41 (s, 12H); ^13^C-NMR (75 MHz, CDCl_3_): δ 199.0, 144.4, 136.8, 129.0, 127.6, 49.5, 36.9; HRMS (ESI^+^) calculated for C_20_H_25_N_2_O_2_: 325.1916 [M + H]^+^, found: 325.1903.

*1,1′-([1,1′-Biphenyl]-4,4′-diyl)bis(2-(piperidin-1-yl)ethanone)* (**13**). Yield, 55%; m.p. 114.3–115.4 °C; ^1^H-NMR (400 MHz, CDCl_3_) δ 8.07 (d, *J* = 7.8, 4H), 7.64 (d, *J* = 7.8, 4H), 3.65 (s, 4H), 3.42 (m, 8H), 1.63 (m, 12H); ^13^C-NMR (75 MHz, CDCl_3_): δ 196.3, 143.4, 136.5, 129.8, 127.1, 65.2, 54.7, 33.0, 22.5; HRMS (ESI+) calculated for C_26_H_33_N_2_O_2_: 405.2542 [M + H]^+^, found: 405.2544.

*1,1′-([1,1′-Biphenyl]-4,4′-diyl)bis(2-morpholinoethanone)* (**14**). Yield, 65%; m.p. 112.2–113.5 °C; ^1^H-NMR (300 MHz, CDCl_3_) δ 8.04 (d, *J* = 8.4, 4H), 7.74 (d, *J* = 8.4, 4H), 3.78 (s, 4H), 3.72 (t, *J* = 4.5, 8H), 2.57 (t, *J* = 4.5, 8H); ^13^C-NMR (125 MHz, CDCl_3_): δ 195.8, 144.7, 135.5, 129.1, 127.7, 67.0, 65.0, 54.1; HRMS (ESI+) calculated for C_24_H_29_N_2_O_4_: 409.2127 [M + H]^+^, found: 409.2123.

*4,4′-Bis(β-diethylamino-propinoyl)-biphenyl* (**15**). Yield, 57%; m.p. 129.2–130.6 °C; ^1^H-NMR (400 MHz, DMSO-*d*_6_) δ 8.15 (d, 4H, *J* = 8.4), 7.99 (d, 4H, *J* = 8.4), 3.71 (t, 4H, *J* = 6.9), 3.43 (t, 4H, *J* = 6.9), 3.22 (q, 8H, *J* = 7.2), 1.270 (t, 12H, *J* = 7.2); ^13^C-NMR (75 MHz, DMSO-*d*_6_): δ 197.2, 144.2, 132.77, 129.0, 127.6, 47.6, 42.0, 33.9, 11.4; HRMS (ESI+) calculated for C_26_H_27_N_2_O_2_: 409.2855 [M + H]^+^, found: 409.2845.

*4,4′-Bis(β-dipropylamino-propinoyl)-biphenyl* (**16**). Yield, 52%; m.p. 131.0–132.6 °C; ^1^H-NMR (400 MHz, CDCl_3_) δ 8.06 (d, 4H, *J* = 8.4), 7.71 (d, 4H, *J* = 8.4), 3.14 (t, 4H, *J* = 6.8), 2.94 (t, 4H, *J* = 6.8), 2.42 (t, 8H, *J* = 7.6), 1.47 (m, 8H), 0.871 (t, 12H, *J* = 7.6); ^13^C-NMR (75 MHz, CDCl_3_): δ 199.7, 144.4, 136.8, 129.0, 127.6, 56.4, 49.5, 36.9, 20.6, 12.1; HRMS (ESI+) calculated for C_30_H_45_N_2_O_2_: 465.3481 [M + H]^+^, found: 465.3479.

*4,4′-Bis(β-azetidin-propinoyl)-biphenyl* (**17**). Yield, 60%; m.p. 150.8–151.1 °C; ^1^H-NMR (400 MHz, CDCl_3_) δ 8.05 (d, 4H, *J* = 8.0), 7.71 (d, 4H, *J* = 8.0), 3.26 (t, 8H, *J* = 6.8), 3.05 (t, 4H, *J* = 7.2), 2.86 (t, 4H, *J* = 7.2), 2.09 (t, 4H, *J* = 6.8); ^13^C-NMR (75 MHz, CDCl_3_): δ 198.4, 144.3, 136.3, 128.8, 127.5, 55.3, 54.5, 37.0, 17.6; HRMS (ESI+) calculated for C_24_H_29_N_2_O_2_: 377.2229 [M + H]^+^, found: 377.2218.

*4,4′-Bis(β-pyrrolidin-propinoyl)-biphenyl* (**18**). Yield, 62%; m.p. 151.2–152.1 °C; ^1^H-NMR (300 MHz, CDCl_3_,) δ 8.06 (d, 4H, *J* = 8.1), 7.72 (d, 4H, *J* = 8.1), 3.28 (t, 4H, *J* = 7.2), 2.97 (t, 4H, *J* = 7.2), 2.62 (m, 8H), 1.824 (m, 8H); ^13^C-NMR (75 MHz, CDCl_3_): δ 198.4, 144.2, 136.3, 128.8, 127.5, 54.3, 51.0, 38.2, 23.5; HRMS (ESI+) calculated for C_26_H_33_N_2_O_2_: 405.2542 [M + H]^+^, found: 405.2535.

*4,4′-Bis(β-(piperidin-propinoyl)-biphenyl* (**19**). Yield, 59%; m.p. 138.5–139.6 °C; ^1^H-NMR (400 MHz, DMSO-*d*_6_) δ 8.15 (d, 4H, *J* = 8.0), 7.99 (d, 4H, *J* = 8.0), 3.66 (t, 4H, *J* = 7.2), 3.55 (t, 4H, *J* = 8.8), 3.44 (t, 4H, *J* = 7.2), 3.01 (t, 4H, *J* = 8.8 ), 1.87 (m, 4H), 1.71 (m, 4H), 1.41 (m, 4H); ^13^C-NMR (125 MHz, CDCl_3_): δ 196.3, 143.4, 135.5, 128.8, 127.4, 52.2, 51.1, 33.0, 22.5, 21.3; HRMS (ESI+) calculated for C_28_H_37_N_2_O_2_ [M + H]^+^: 433.2855, found: 433.2844.

*4,4′-Bis(β-(morpholino-propinoyl)-biphenyl* (**20**). Yield, 58%; m.p. 140.1–141.0 °C; ^1^H-NMR (400 MHz, CDCl_3_) δ 8.06 (d, *J* = 8.0, 4H), 7.72 (d, *J* = 8.0, 4H), 3.76 (m, 8H), 3.22 (t, *J* = 7.2, 4H), 2.86 (t, *J* = 7.2, 4H), 2.53 (m, 8H); ^13^C-NMR (125 MHz, CDCl_3_): δ 198.4, 144.6, 136.4, 129.0, 127.7, 66.9, 53.9, 53.7, 36.1; HRMS (ESI+) calculated for C_26_H_33_N_2_O_4_: 437.2440 [M + H]^+^, found: 437.2431.

*4,4′-Bis(β-azepan-propinoyl)-biphenyl* (**21**). Yield, 52%; m.p. 109.2–111.5 °C; ^1^H-NMR (400 MHz, CDCl_3_) δ 8.05 (d, 4H, *J* = 8.4), 7.70 (d, 4H, *J* = 8.4), 3.23 (t, 4H, *J* = 7.6), 3.03 (t, 4H, *J* = 7.6), 2.74 (t, 8H, *J* = 5.6), 1.68 (m, 8H), 1.59 (m, 8H); ^13^C-NMR (75 MHz, CDCl_3_): δ 199.1, 144.4, 136.5, 128.9, 127.6, 55.6, 53.3, 37.0, 27.7, 27.1; HRMS (ESI+) calculated for C_30_H_41_N_2_O_2_: 461.3168 [M + H]^+^, found: 461.3158.

*4,4′-Bis(β-4-methylpiperazin-propinoyl)-biphenyl* (**22**). Yield, 50%; m.p. 146.1–148.7 °C; ^1^H-NMR (400 MHz, CDCl_3_) *δ* 8.06 (d, 4H, *J* = 8.4), 7.73 (d, 4H, *J* = 8.4), 3.23 (t, 4H, *J* = 7.6), 2.89 (t, 4H, *J* = 7.6), 2.59 (m, 8H), 2.48 (m, 8H), 2.31 (s, 6H); ^13^C-NMR (75 MHz, CDCl_3_): δ 198.6, 144.3, 136.4, 128.7, 127.5, 55.1, 53.2, 53.1, 46.0, 36.4; HRMS (ESI+) calculated for C_28_H_39_N_4_O_2_ [M + H]^+^: 463.3073, found: 463.3067.

*1,1′-([1,1′-Biphenyl]-4,4′-diyl)bis(4-(piperidin-1-yl)butan-1-one)* (**23**). Yield, 53%; m.p. 146.4–147.8 °C; ^1^H-NMR (400 MHz, CDCl_3_) δ 8.12 (d, 4H, *J* = 8.0), 7.78 (d, 4H, *J* = 8.0), 3.09 (t, 4H, *J* = 7.2), 2.44 (m, 12H), 2.07 (m, 4H), 1.59 (m, 8H), 1.44 (m, 4H); ^13^C-NMR (125 MHz, CDCl_3_): δ 199.7, 144.4, 136.6, 129.0, 127.6, 58.5, 54.5, 36.7, 25.7, 24.4, 21.5. HRMS (ESI+) calculated for C_30_H_41_N_2_O_2_ [M + H]^+^: 461.3168, found: 461.3162.

*1,1′-([1,1′-Biphenyl]-4,4′-diyl)bis(5-(piperidin-1-yl)pentan-1-one)* (**24**). Yield, 48%; m.p. 115.7–117.8 °C; ^1^H-NMR (400 MHz, CDCl_3_) δ 8.05 (d, 4H, *J* = 8.4), 7.70 (d, 4H, *J* = 8.4), 3.03 (t, 4H, *J* = 7.2), 2.45 (m, 12H), 1.80 (m, 4H), 1.59 (m, 12H), 1.44 (m, 4H); ^13^C-NMR (125 MHz, CDCl_3_): δ 199.7, 144.4, 136.6, 129.0, 127.6, 58.5, 54.5, 36.7, 25.7, 24.4, 21.5; HRMS (ESI+) calculated for C_32_H_45_N_2_O_2_ [M + H]^+^: 489.3481; found, 489.3473.

*1,1′-(Ethane-1,2-diylbis(4,1-phenylene))bis(2-(dimethylamino)ethanone)* (**25**). Yield, 58%; m.p. 104.2–106.9 °C; ^1^H-NMR (300 MH_Z_, CDCl_3_) δ 7.91 (d, 4H, *J* = 8.1), 7.22 (d, 4H, *J* = 8.1), 3.75 (s, 4H), 2.99 (s, 4H), 2.39 (s, 12H). ^13^C-NMR (125 MHz, CDCl_3_): δ 196.2, 147.2, 134.2, 130.0, 128.9, 65.3, 45.9, 37.5; HRMS (ESI+): calculated for C_22_H_29_N_2_O_2_ [M + H]^+^: 353.2229, found: 353.2217.

*1,1′-(Ethane-1,2-diylbis(4,1-phenylene))bis(2-(piperidin-1-yl)ethanone)* (**26**). Yield, 52%; m.p. 96.7–98.6 °C; ^1^H-NMR (300 MHz, CDCl_3_) *δ* 7.92 (d, 4H, *J* = 7.8), 7.21 (d, 4H, *J* = 7.8), 3.79 (s, 4H), 2.99 (s, 4H), 2.58 (m, 8H), 1.67 (m, 8H), 1.47 (m, 4H); ^13^C-NMR (125 MHz, CDCl_3_): δ 196.7, 147.0, 134.6, 128.8, 128.6, 65.5, 55.1, 37.6, 26.0, 24.2; HRMS (ESI+) calculated for C_28_H_37_N_2_O_2_ [M + H]^+^, 433.2855, found: 433.2850.

*1,1′-(Ethane-1,2-diylbis(4,1-phenylene))bis(2-(4-methylpiperazin-1-yl)ethanone)* (**27**). Yield, 54%; m.p. 150.9–152.2 °C; ^1^H-NMR (300 MHz, CDCl_3_) δ 7.91 (d, 4H, *J* = 8.1), 7.223 (d, 4H, *J* = 8.1), 3.79 (s, 4H), 2.99 (s, 4H), 2.64 (m, 8H), 2.530 (m, 8H) 2.31 (s, 6H); ^13^C-NMR (125 MHz, CDCl_3_): δ 195.9, 147.1, 134.3, 128.9, 128.5, 64.4, 55.0, 53.4, 46.0, 37.5; HRMS (ESI+) calculated for C_28_H_39_N_4_O_2_ [M + H]^+^: 463.3073, found: 463.3066.

*1,1′-(Ethane-1,2-diylbis(4,1-phenylene))bis(5-morpholinopentan-1-one)* (**28**). Yield, 52%; m.p. 160.1–162.6 °C; ^1^H-NMR (300 MHz, CDCl_3_) δ 7.91 (d, 4H, *J* = 8.4), 7.23 (d, 4H, *J* = 8.4), 3.79 (m, 12H), 3.01 (s, 4H), 2.63 (m, 8H); ^13^C-NMR (125 MHz, CDCl_3_): δ 195.8, 147.2, 134.3, 128.9, 128.5, 67.0, 64.8, 54.1, 37.5; HRMS (ESI+) calculated for C_28_H_33_N_2_O_4_ [M + H]^+^: 437.2440, found: 437.2434.

*1,1′-(Ethane-1,2-diylbis(4,1-phenylene))bis(3-(dimethylamino)propan-1-one)* (**29**). Yield, 56%; m.p. 94.5–95.6 °C; ^1^H-NMR (400 MHz, CDCl_3_) δ 7.91 (d, 4H, *J* = 8.0 Hz), 7.25 (d, 4H, *J* = 8.0), 3.14 (t, 4H, *J* = 7.2), 2.99 (s, 4H), 2.77 (t, 4H, *J* = 7.2), 2.24 (s, 12H); ^13^C-NMR (125 MHz, CDCl_3_): δ 198.6, 147.0, 135.1, 129.0, 128.5, 54.4, 45.6, 37.5, 36.8; HRMS (ESI+) calculated for C_24_H_33_N_2_O_2_ [M + H]^+^: 381.2545, found: 381.2542.

*1,1′-(Ethane-1,2-diylbis(4,1-phenylene))bis(3-(piperidin-1-yl)propan-1-one)* (**30**). Yield, 52%; m.p. 103.5–104.8 °C; ^1^H-NMR (400 MHz, CDCl_3_) δ 7.86 (d, *J* = 8.2, 4H), 7.21 (d, *J* = 8.2, 4H), 3.10 (m, 4H), 2.99 (s, 4H), 2.79 (t, *J* = 7.5, 4H), 2.45 (s, 8H), 1.60 (m, 8H), 1.44 (d, *J* = 4.8, 4H); ^13^C-NMR (125 MHz, CDCl_3_): δ 199.0, 146.9, 135.2, 129.0, 128.5, 54.8, 54.0, 37.5, 36.3, 26.0, 24.3; HRMS (ESI+) calculated for C_30_H_40_N_2_O_2_ [M + H]^+^: 461.3168, found: 461.3162.

*1,1′-(Ethane-1,2-diylbis(4,1-phenylene))bis(3-morpholinopropan-1-one)* (**31**). Yield, 56%; m.p. 105.2–106.6 °C; ^1^H-NMR (400 MHz, CDCl_3_) δ 7.86 (d, *J* = 8.2, 4H), 7.22 (d, *J* = 8.1, 4H), 3.75–3.66 (m, 8H), 3.17 (t, *J* = 7.3, 4H), 3.00 (s, 4H), 2.84 (t, *J* = 7.3, 4H), 2.53 (s, 8H); ^13^C-NMR (125 MHz, CDCl_3_): δ 198.7, 147.0, 135.2, 129.0, 128.5, 67.1, 53.9 53.8, 37.5, 36.1; HRMS (ESI+) calculated for C_28_H_36_N_2_O_4_ [M + H]^+^: 465.2753, found: 465.2744.

*1,1′-(Ethane-1,2-diylbis(4,1-phenylene))bis(4-(piperidin-1-yl)butan-1-one)* (**32**). Yield, 53%; m.p. 145.2–146.6 °C; ^1^H-NMR (400 MHz, CDCl_3_) δ 7.87 (d, 4H, *J* = 8.4), 7.21 (d, 4H, *J* = 8.4), 2.99 (s, 4H), 2.95 (t, 4H, *J* = 7.2), 2.32 (m, 12H), 1.86 (m, 4H), 1.48 (m, 8H), 1.35 (m, 4H); ^13^C-NMR (125 MHz, CDCl_3_): δ 199.9, 146.7, 135.5, 129.0, 128.5, 58.7, 54.6, 37.5, 36.6, 25.9, 24.5, 21.6; HRMS (ESI+) calculated for C_32_H_45_N_2_O_2_ [M + H]^+^, 488.3481, found: 488.3475.

*1,1′-(Ethane-1,2-diylbis(4,1-phenylene))bis(5-(piperidin-1-yl)pentan-1-one)* (**33**). Yield, 68%; m.p. 146.1–147.5 °C; ^1^H-NMR (300 MHz, CDCl_3_) δ 7.87 (d, 4H, *J* = 8.1 Hz), 7.21 (d, 4H, *J* = 8.1), 3.00 (s, 4H), 2.97 (t, 4H, *J* = 7.2), 2.36 (m, 12H), 1.74 (m, 4H), 1.60 (m, 12H), 1.44 (m, 4H); ^13^C-NMR (125 MHz, CDCl_3_): δ 200.1, 146.7, 135.3, 128.9, 128.5, 59.2, 54.7, 38.5, 37.5, 26.6, 25.9, 24.5, 22.6; HRMS (ESI+) calculated for C_34_H_49_N_2_O_2_ [M + H]^+^: 517.3794, found: 517.3787.

#### 3.1.3. General Method for Synthesis of Target Compounds (**34**–**36**)

To a stirred solution of compounds (**19**, **20** and **30**) (1.0 mmol) in anhydrous THF (30 mL), sodium borohydride (3.0 mmol) was added with stirring. The reaction mixture was stirred at 50 °C for 6 h. Then the reaction mixture was filtered and quenched by methanol. After the concentrated, the residue was subjected to silica gel column chromatography with DCM:MeOH = 100:1 as an eluent to afford the target alcohols (**34**–**36**) with the yield of 52%–57%.

*1,1′-([1,1′-Biphenyl]-4,4′-diyl)bis(3-(piperidin-1-yl)propan-1-ol)* (**34**). Yield, 52%; m.p. 139.5–141.6 °C; ^1^H-NMR (400 MHz, CDCl_3_) δ 7.57 (d, 4H, *J* = 8.4), 7.43 (d, 4H, *J* = 8.4), 4.98 (t, 2H, *J* = 5.6), 2.58 (m, 8H), 2.43 (m, 4H), 1.87 (m, 4H), 1.64 (m, 8H), 1.06 (m, 4H); ^13^C-NMR (75 MHz, CDCl_3_): δ 144.1, 139.6, 126.8, 125.9, 75.5, 57.8, 54.7, 33.6, 26.1, 24.3; HRMS (ESI+) calculated for C_28_H_41_N_2_O_2_ [M + H]^+^: 437.3168, found: 437.3162.

1*,1′-([1,1′-Biphenyl]-4,4′-diyl)bis(3-morpholinopropan-1-ol)* (**35**). Yield, 57%; m.p. 131.2–133.6°C; ^1^H-NMR (400 MHz, CDCl_3_) δ 7.57 (d, 4H, *J* = 7.6), 7.43 (d, 4H, *J* = 7.6), 6.40 (brs, 2H), 4.96 (brs, 2H), 3.75 (m, 8H), 2.65 (m, 8H), 2.49 (m, 4H), 1.89 (m, 4H, *J* = 4.4 ); ^13^C-NMR (75 MHz, CDCl_3_): δ 143.6, 139.5, 126.7, 125.8, 75.0, 66.8, 57.3, 53.5, 33.3; HRMS (ESI+) calculated for C_28_H_37_N_2_O_4_ [M + H]^+^: 440.2753, found: 440.2743.

*1,1′-(Ethane-1,2-diylbis(4,1-phenylene))bis(3-(piperidin-1-yl)propan-1-ol)* (**36**). Yield, 52%; m.p. 141.5–142.6 °C; ^1^H-NMR (400 MHz, CDCl_3_): δ 7.28 (d, 4H, *J* = 8.4), 7.15 (d, 4H, *J* = 8.4), 4.90 (t, 2H, *J* = 5.6) 2.89 (s, 4H), 2.57 (m, 8H), 2.41 (m, 4H), 1.83 (m, 4H), 1.62 (m, 8H), 1.46 (m, 4H); ^13^C-NMR (75 MHz, CDCl_3_): δ 142.7, 140.3, 128.2, 125.5, 75.5, 57.7, 54.6, 37.6, 33.6, 26.0, 24.2; HRMS (ESI+) calculated for C_30_H_45_N_2_O_2_ [M + H]^+^: 465.3481, found: 465.3462.

### 3.2. Biological Evaluation

All of the assay of acetylcholinesterase (AChE) and butyrylcholinesterase (BuChE) were performed according to the method described by Ellman et al. [17] using the rat AChE purified from the rat brain and human BuChE purified by human serum. In an enzymatic reaction medium contained different concentration of tested compounds and AChE suspended in the phosphate buffer solution at pH 7.2. After 30 min at room temperature, acetylthiocholine iodide and 5.5′-dithiobis-2-nitrobenzoic acid (DNTB) were added and the reaction mixture incubated at 37 °C for 60 min. After the initiation of enzymatic reaction, absorbance of each tested compound mixture of colored end-product was measured by using the UV-VIS spectrophotometer at 412 nm. The parallel control experiment was carried without tested compounds in the reaction mixture. For BuChE inhibitory assay, the procedure described above was followed except for the use of enzyme and substrate, instead of which butyrylcholinesterase and butyrylthiocholine chloride were used, respectively. Assays were done with a blank containing all compounds except AChE or BuChE in order to account for nonenzymatic reactions. Each inhibitory test was conducted in triplicate. Donepezil was used as a positive control of AChE and ISO-OMPA was used as a positive control of BuChE, respectively. Each concentration was assayed in triplicate and data were expressed as the mean ± SEM.

### 3.3. Molecular Docking

To further investigate the interaction of the most active compound **19** for AChE and BuChE, a molecular docking study was performed by utilizing the Gold 3.0.1 software package (Cambridge Crystallographic Data Center, Cambridge, UK). The X-ray crystal structure of the AChE (PDB code: 1EVE) and BuChE (PDB code: 4BDS) were obtained from protein data bank. Discovery Studio and Pymol program were also used to prepare 2D and 3D schematic diagrams of docking model to exhibit different interaction types between **19** and AChE/BuChE.

## 4. Conclusions

In summary, a new class of diphenyl/dibenzyl derivatives (**12**–**36**) has been designed, synthesized, and determined to function as novel dual inhibitors of AChE and BuChE. Most of these compounds possessed moderate to high AChE and BuChE inhibitory activities in vitro. Compound **19** showed the most potent AChE inhibitory activity, in the sub-micromolar range (IC_50_ = 0.096 µM), and moderate BuChE inhibition, with an IC_50_ value of 1.25 µM. Compound **15** exhibited the most potent inhibition against BuChE, with an IC_50_ value of 0.74 µM, which was as potent as the reference compound iso-OPMA. Our molecular modeling study confirmed that compound **19** is capable of binding to the active-site cavity of both AChE and BuchE. Among the tested compounds, compound **19** is considered to be a promising dual inhibitor against AChE and BuChE and, thus, a therapeutic candidate in AD.

## Figures and Tables

**Table 1:**
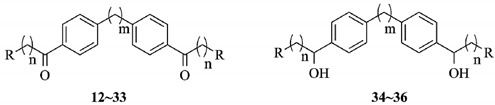
AChE and BuChE inhibition by target compounds **12**–**36**.

Compounds ^a^	m	n	R	IC_50_ µM ± SEM	
				AChE ^b^ ± SEM	BuChE ^c^ ± SEM
**12**	0	1	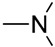	^d^	91.68 ± 15.68
**13**	0	1	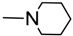	^d^	71.68 ± 3.01
**14**	0	1	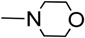	^d^	1.44 ± 0.23
**15**	0	2	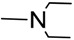	1.18 ± 0.29	0.74 ± 0.09
**16**	0	2	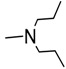	0.88 ± 0.17	2.83 ± 0.50
**17**	0	2	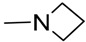	0.71 ± 0.06	2.39 ± 0.75
**18**	0	2	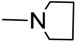	0.33 ± 0.06	1.16 ± 0.09
**19**	0	2	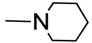	0.096 ± 0.006	1.25 ± 0.15
**20**	0	2	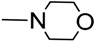	0.97 ± 0.08	0.72 ± 0.10
**21**	0	2	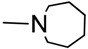	1.43 ± 0.33	1.94 ± 0.07
**22**	0	2	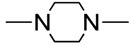	1.61 ± 0.38	13.77 ± 0.81
**23**	0	3	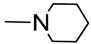	1.32 ± 0.43	5.9 ± 0.40
**24**	0	4	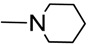	0.82 ± 0.16	2.65 ± 0.11
**25**	2	1	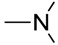	62.27 ± 14.46	9.11 ± 2.55
**26**	2	1	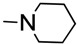	74.54 ± 8.50	20.72 ± 5.44
**27**	2	1	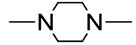	74.13 ± 11.53	66.51 ± 17.04
**28**	2	1	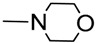	^d^	6.30 ± 1.60
**29**	2	2	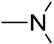	2.62 ± 0.63	1.21 ± 0.22
**30**	2	2	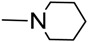	0.50 ± 0.06	3.14 ± 0.24
**31**	2	2	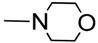	3.53 ± 0.79	6.38 ± 1.26
**32**	2	3	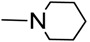	3.67 ± 0.92	19.18 ± 4.78
**33**	2	4	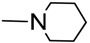	0.19 ± 0.08	1.64 ± 0.27
**34**	0	2	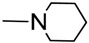	31.74 ± 10.24	13.78 ± 1.59
**35**	0	2	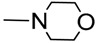	^d^	^d^
**36**	2	2	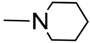	74.93 ± 35.56	25.94 ± 6.81
Donepezil				0.044	
ISO-OMPA					0.72

SEM: Standard error of the mean. ^a^ The structure of compounds **12**–**36** shown in Scheme 1. ^b^ 50% inhibitory concentration (means ± SEM of three experiments using AChE from rat brain). ^c^ 50% inhibitory concentration (means ± SEM of three experiments using BuChE from human serum). ^d^ Concentrations of the compounds up to 100 µM/L were inactive.
